# Epigenetic Suppression of RASAL1 by HDAC3 and Cofactor YY1 Promotes Fibroblast–Myofibroblast Transition and Renal Fibrosis

**DOI:** 10.34133/research.1073

**Published:** 2026-01-29

**Authors:** Fang Chen, Lijun Zhang, Weiying Liu, Bingbing Zhang, Shuren Wang, Zhengdong Zhou, Wei Wang, Jiansong Shen, Yijun Deng, Wangsen Cao

**Affiliations:** ^1^ Yancheng Key Laboratory of Molecular Epigenetics, Yancheng Medical Research Center of Nanjing University Medical School, The First People’s Hospital of Yancheng, Yancheng 224006, China.; ^2^ Department of Urology, The First People’s Hospital of Yancheng, The Yancheng Clinical College of Xuzhou Medical University, Yancheng 224006, China.; ^3^ Jiangsu Key Lab of Molecular Medicine, Nanjing University Medical School, Nanjing 210093, China.; ^4^ Department of Critical Care Medicine, The First People’s Hospital of Yancheng, The Yancheng Clinical College of Xuzhou Medical University, Yancheng 224006, China.; ^5^ Department of Gastroenterology, The First People’s Hospital of Shangqiu, Shangqiu 476005, China.; ^6^ Department of Pathology, The First People’s Hospital of Yancheng, The Yancheng Clinical College of Xuzhou Medical University, Yancheng 224006, China.; ^7^ Department of Nephrology, The First People’s Hospital of Yancheng, The Yancheng Clinical College of Xuzhou Medical University, Yancheng 224006, China.; ^8^ Yangzhou Precision Research Institute of Kidney Disease, Yangzhou Key Lab of Kidney Diseases, Department of Nephrology, Northern Jiangsu People’s Hospital, Yangzhou 225001, China.

## Abstract

Fibroblast–myofibroblast transition (FMT) and the resultant renal fibrosis are central pathological features of chronic kidney disease (CKD). Epigenetic suppression of RASAL1 (Ras protein activator like 1), an antifibrotic regulator in fibroblasts, is a key driver of this process. However, the underlying mechanisms are only partially understood. Here, we identify histone deacetylase 3 (HDAC3) as a critical epigenetic suppressor of RASAL1 expression in FMT of renal fibrosis. In mouse models of renal fibrosis induced by unilateral ureteral obstruction and aristolochic acid I, RASAL1 suppression coincided with a preferential increase in HDAC3. Fibroblast-specific *Hdac3* knockout mice exhibited preserved RASAL1 expression, attenuated FMT, and reduced renal fibrosis compared to wild-type controls. Consistently, pharmacological inhibition of HDAC3 with RGFP966 similarly restored RASAL1 expression, inhibited FMT, and alleviated renal fibrotic pathology. In cultured renal fibroblasts, HDAC3 overexpression or inhibition by RGFP966 inversely affected RASAL1 abundance and major FMT parameters, which was coregulated by the repressive transcriptional factor YY1 (Yin Yang 1). Notably, targeted silencing of RASAL1 abrogated the antifibrotic effects of HDAC3 inhibition both in vitro and in vivo, underscoring the functional significance of the HDAC3–YY1–RASAL1 axis in FMT and fibrogenesis. Given that FMT is a conserved feature of fibrotic diseases across multiple organs, restoring RASAL1 expression via HDAC3 and YY1 modulation offers promising therapeutic strategies for CKD and potentially broader fibrotic disorders.

## Introduction

Renal fibrosis represents a common histopathological feature of chronic kidney disease (CKD) of all etiologies, including glomerulonephritis, hypertensive nephropathy, and diabetic nephropathy [1]. CKD has a global prevalence of over 10%, with increasing morbidity and mortality [2], for which effective therapies are currently lacking [3]. Pathologically, renal fibrosis reflects a maladaptive repair response following repetitive kidney damage. In contrast to the self-restrained nature of physiological wound healing, fibrosis is characterized by persistent injury to renal cells, perpetuated fibroblast activation, and progressive accumulation of myofibroblasts. These myofibroblasts secrete excessive amounts of nondegradable extracellular matrix proteins, such as type 1 collagen (Colla 1) and fibronectin, resulting in scar formation and structural disruption of the kidney [4]. Myofibroblasts are the principal effector cells for renal fibrosis and may arise via transdifferentiation from renal epithelia, endothelia, pericytes, and infiltrating macrophages [5] or originate directly from resident fibroblasts through fibroblast–myofibroblast transition (FMT) [6]. It is estimated that approximately 50% of myofibroblasts in renal fibrotic lesions derive from intrinsic renal fibroblasts [7]. These myofibroblasts display cellular immortality, maintaining proliferative capacity and fibroblast-like phenotypes across multiple in vitro passages [8]. Therefore, perpetuated FMT driven by stable epigenetic alterations constitutes a fundamental mechanism underlying the initiation and progression of renal fibrosis.

RASAL1 (Ras protein activator like 1) is a calcium-dependent Ras–guanosine triphosphatase-activating protein predominantly expressed in renal fibroblasts. It promotes the conversion of Ras–guanosine triphosphate to inactive Ras–guanosine diphosphate, thereby suppressing Ras signaling—a pathway causally involved in renal fibrosis [9,10]. Ras proteins function as molecular switches that transmit signals from membrane receptors to downstream effectors, regulating diverse cellular processes including cell proliferation, differentiation, migration/adhesion, and apoptosis. These signaling cascades primarily involve the mitogen-activated protein kinase/extracellular signal–regulated kinase, phosphatidylinositol 3-kinase/AKT/mechanistic target of rapamycin, and transforming growth factor-β (TGFβ)/Smad pathways. Loss of RASAL1 leads to sustained RAS activation, which, in turn, drives persistent fibroblast proliferation, FMT, and extracellular matrix deposition [11,12]. Transgenic mice overexpressing RASAL1 display reduced fibroblast proliferation and milder kidney fibrotic injury following unilateral ureteral obstruction (UUO) [13], whereas Rasal1 knockout mice subjected to UUO exhibit increased fibroblast activation and exacerbated renal fibrosis compared to wild-type controls [14], suggesting that RASAL1 is a critical regulator of FMT and renal fibrosis. RASAL1 expression is suppressed in fibrotic kidneys, primarily due to promoter hypermethylation induced by DNA methyltransferase DNMT1 [12]. This epigenetic suppression is executed by a transcriptional repressor complex comprising coregulatory factors, repressor proteins, and histone deacetylases (HDACs) [15]. Notably, RASAL1 suppression during renal fibroblast activation is associated with significant increase in HDAC1, HDAC3, and HDAC4 [16]. However, the precise HDAC subtype responsible for driving RASAL1 suppression and promoting FMT remains unidentified.

HDAC aberration affects renal fibrosis through diverse targets and signaling pathways across multiple renal cell types [17]. Our previous investigations demonstrated that HDAC3 is preferentially up-regulated in mouse fibrotic kidneys [18–20]. As a member of class I HDAC family, HDAC3 serves as a pivotal transcriptional regulator through its involvement in chromatin remodeling, histone deacetylation, transcription factor interaction, and recruitment of corepressor complex [21], leading to chromatin compaction and gene transcriptional silencing [22]. HDAC3 interacts with several transcription factors, including YY1 (Yin Yang 1), NF-κB (nuclear factor κB), FOXM1 (forkhead box M1), and FOXO3 (forkhead box O3) [23,24], to modulate their transcriptional output and downstream target gene expression. In addition, HDAC3 associates with corepressor complexes such as nuclear receptor corepressor and silencing mediator of retinoid and thyroid hormone receptors [25,26] to reinforce transcriptional repression. These multifaceted roles underscore HDAC3 as a key epigenetic regulator of transcriptional inhibition. However, its specific contribution to DNA-methylation-mediated RASAL1 suppression and FMT in renal fibrosis remains to be elucidated.

We hypothesized that HDAC3 plays a critical role in repressing RASAL1 expression during FMT and the progression of renal fibrosis. To investigate this, we generated a fibroblast-specific *Hdac3* conditional knockout mouse line and used both genetic and pharmacological interventions across 2 distinct murine models of renal fibrosis—UUO and AAI (aristolochic acid I). Our findings provide compelling evidence that HDAC3-mediated transcriptional repression of RASAL1 promotes FMT, suggesting that HDAC3 may serve as a therapeutic target for fibrotic diseases affecting kidney and extrarenal organs.

## Results

### UUO and AAI kidneys display inverse HDAC3 elevation and RASAL1 suppression in renal fibroblasts

**Fig. 1.**
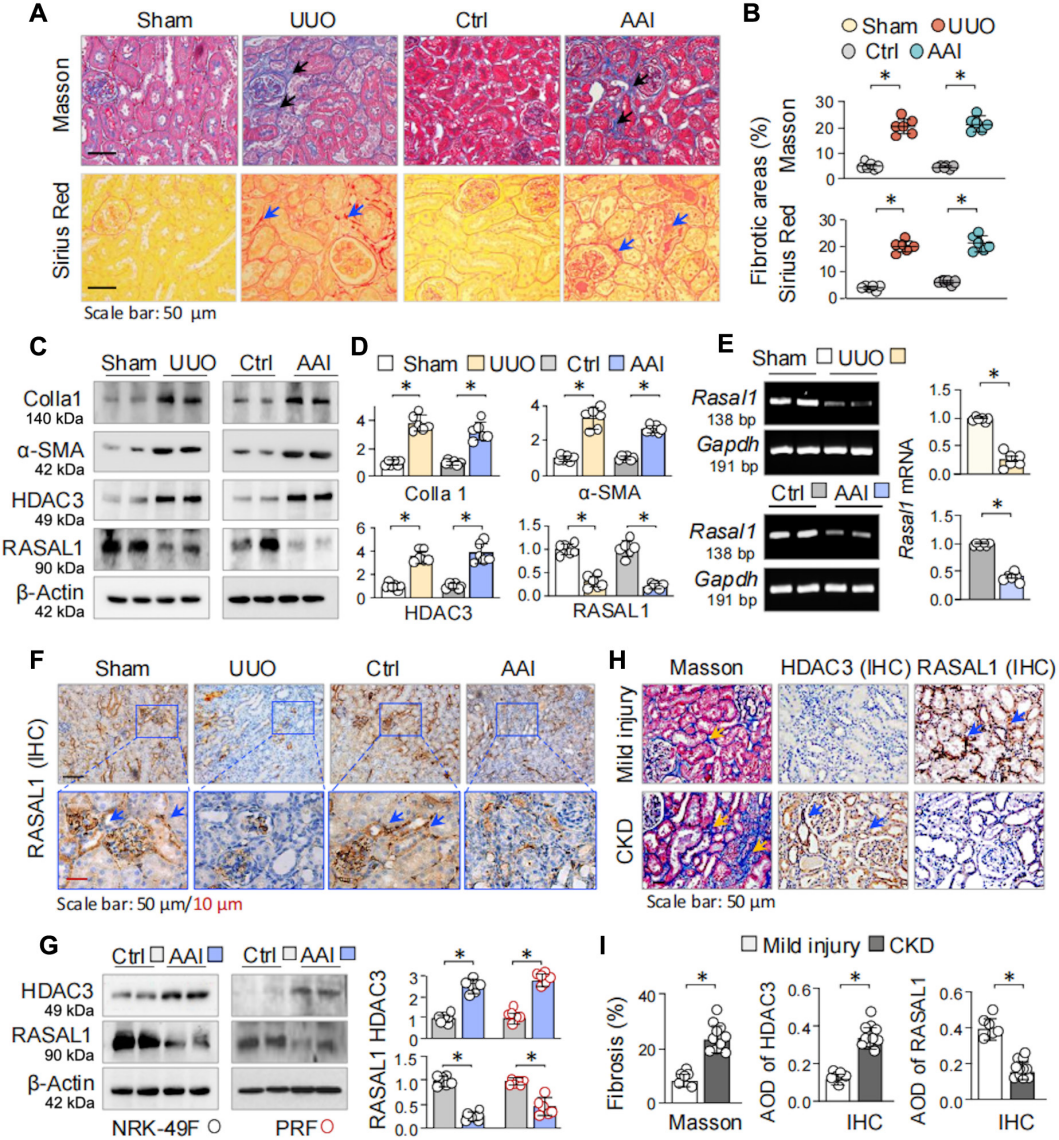
Fibrotic kidneys induced by UUO or AAI display aberrant up-regulation of HDAC3 and suppression of RASAL1 in renal fibroblasts. C57BL/6 mice were subjected to UUO for 7 d or AAI (5 mg/kg, intraperitoneal injection every other day) for 14 d (6 mice in each group). (A) Representative photomicrographs of kidney sections stained with Masson’s trichrome and Sirius Red from sham-, UUO-, control-, and AAI-treated mice. The arrows indicate collagen-stained fibrotic areas. (B) Quantitation of renal fibrosis in (A). (C) Western blots of renal tissues for Colla 1, α-SMA, HDAC3, and RASAL1. Two representative samples from each group were shown. β-Actin served as loading control. (D) Quantifications of (C). (E) RT-PCR of renal tissues from sham-, UUO-, control-, and AAI-treated mice (*n* = 6) for *Rasal1* mRNAs. *Gapdh* served as the internal control. Two random samples from each group were shown. The right panel was the quantification. bp, base pairs. Data were presented as means ± SEM based on 6 renal samples. *P = 0.05, 2-tailed unpaired *t* test for (B), (D), and (E). (F) Representative photomicrographs of kidney sections from sham-, UUO-, control-, and AAI-treated mice stained for RASAL1 by IHC staining. Positive staining was indicated by arrows. (G) Western blotting. NRK-49F and mouse primary renal fibroblast (PRF) cells were treated with AAI (30 μM) for 24 h, and then the cell lysates were tested for HDAC3 and RASAL1. The quantitation was on the right side. Data were presented as means ± SD of 6 repeated cell assays. *P < 0.05, 2-tailed unpaired *t* test. (H) Representative photomicrographs of kidney sections from renal patients (mild injury and CKD) stained by Masson’s trichrome for renal fibrosis and IHC for HDAC3 and RASAL1. The arrows indicate collagen-stained fibrotic areas and positively stained fibroblast-rich areas. (I) Quantifications of (H) presented as means ± SEM of 6 mild injury and 10 CKD samples. Data were presented as means ± SEM based on 6 renal samples. *P < 0.05, 2-tailed unpaired *t* test.

To explore regulatory mechanisms underlying fibroblast activation and renal fibrosis, we used 2 well-known mouse models: the UUO model, characterized by severe tubular injury and fibrosis due to urinary tract obstruction, and the AAI sodium salt model, which induces progressive renal tubular atrophy and interstitial fibrosis [27,28]. Renal tissues were examined at d7 post-UUO and d14 after AAI exposure. Histological assessment revealed marked renal tubular injury and fibrotic lesions in both models (Fig. 1A, indicated by arrows), evidenced by Masson’s trichrome staining (5.07 ± 0.46% of sham versus 20.43 ± 1.23% of UUO and 4.82 ± 0.18% of control versus 21.41 ± 1.13% of AAI, *P < 0.05; top panels of Fig. 1A and B) and Sirius Red staining (3.97 ± 0.24% of sham versus 19.95 ± 0.95% of UUO and 6.15 ± 0.28% of control versus 20.96 ± 1.25% of AAI, *P < 0.05; bottom panel of Fig. 1A and B). Western blot analyses demonstrated a progressive increase in the myofibroblast marker α-smooth muscle actin (α-SMA) and extracellular matrix protein Colla 1 following UUO or AAI treatment. Notably, HDAC3 expression was markedly increased, while the antifibrotic protein RASAL1 was down-regulated (Fig. 1C and D). Reverse transcription polymerase chain reaction (RT-PCR) confirmed a substantial reduction in *Rasal1* mRNA levels following UUO or AAI treatment (Fig. 1E). Immunohistochemistry (IHC) staining of mouse kidney sections revealed that RASAL1 expression was predominantly localized to areas enriched in renal fibroblasts, consistent with previous studies demonstrating constitutive high expression of RASAL1 in quiescent renal fibroblasts [12,14]. However, its expression was markedly diminished after UUO or AAI treatment (Fig. 1F). Further in vitro studies using mouse primary renal fibroblasts and renal fibroblast NRK-49F cells treated with AAI corroborated these findings. AAI treatment led to significant HDAC3 up-regulation and concomitant RASAL1 suppression (Fig. 1G and Fig. S1A and B).

To validate these findings in human samples, we examined renal biopsies from 16 patients: 6 with proteinuria (mild fibrotic renal injury) and 10 with CKD of various etiologies. IHC staining revealed low HDAC3 and high RASAL1 expression in the proteinuria group, whereas CKD samples displayed elevated HDAC3 and diminished RASAL1 levels (Fig. 1H and I), consistent with observations in the mouse models. Collectively, these results demonstrate that FMT and renal fibrogenesis are associated with elevated HDAC3 and reduced RASAL1 expression in renal fibroblasts across experimental mouse models and human pathology.

### Loss of HDAC3 attenuates RASAL1 suppression and renal fibrosis

**Fig. 2.**
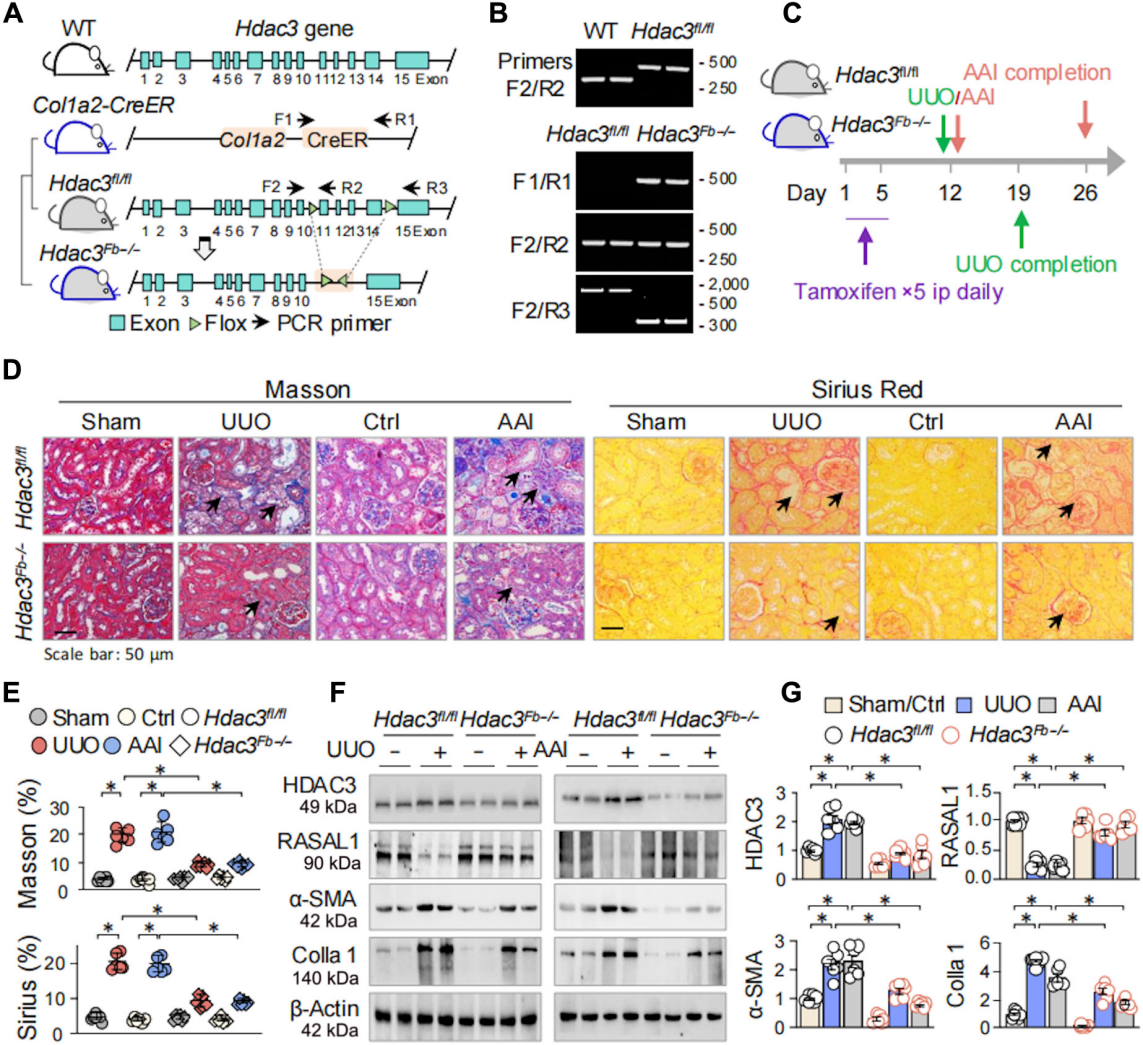
HDAC3 knockout resists RASAL1 suppression and renal fibrosis in fibrotic mice. (A) Schematic diagram of the construction of fibroblast-specific *Hdac3* knockout mice (*Hdac3^fl/fl^-Col1a2*-CreER, *Hdac3^Fb−/−^*). Black arrows indicate the location of genotyping primers. (B) Genotyping of *Hdac3* wild-type (WT), *Hdac3^fl/fl^* (top), and conditional *Hdac3^Fb−/−^* mice (bottom). (C) Schematic diagram of the animal experiment design and process. ip, intraperitoneally. (D) *Hdac3^fl/fl^* and *Hdac3^Fb−/−^* mice were subjected to sham or UUO for 7 d or to control or AAI for 14 d, 6 mice per group. Left: Representative photomicrographs of kidney sections stained with Masson’s trichrome from all groups. Right: Representative photomicrographs of kidney sections stained with Sirius Red from all groups. The black arrows indicate collagen-stained fibrotic areas. (E) Quantifications of renal fibrosis stained with Masson’s trichrome and Sirius Red from the experimental mice indicated above. (F) Western blotting. The renal tissue homogenates were assayed for HDAC3, RASAL1, α-SMA, Colla 1, and β-actin, which served as the internal control. Two randomly selected samples from each group were shown. (G) Quantifications of (F). Data were presented as means ± SEM based on 6 renal samples. *P < 0.05, 2-way ANOVA.

To delineate the functional contribution of HDAC3 dysregulation to FMT and renal fibrosis, we generated a strain of tamoxifen-inducible, fibroblast-specific *Hdac3* knockout (*Hdac3^Fb−/−^*) mice (see Materials and Methods and Fig. 2A) and confirmed successful recombination by genotyping (Fig. 2B). Both control *Hdac3* floxed (*Hdac3^fl/fl^*) and *Hdac3^Fb−/−^* mice received intraperitoneal tamoxifen injection for 5 consecutive days, followed by a 7-d waiting period before UUO or AAI treatment (Fig. 2C). Histological analysis revealed substantially reduced fibrotic lesions in *Hdac*3*^Fb−/−^* mice after UUO or AAI treatment compared to control *Hdac3^fl/fl^* counterparts. Masson’s trichrome staining quantified significantly less collagen deposition in *Hdac*3*^Fb−/−^* mice (9.33 ± 0.49% of *Hdac3^Fb−/−^/*UUO versus 19.01 ± 0.73% of *Hdac3^fl/fl^*/UUO mice and 9.90 ± 0.57% of *Hdac3^Fb−/−^*/AAI versus 19.94 ± 0.78% of *Hdac3^fl/fl^*/AAI mice, *P < 0.05; Fig. 2D and E), and the results from Sirius Red staining (8.98 ± 0.54% of *Hdac3^Fb−/−^*/UUO versus 20.37 ± 0.74% of *Hdac3^fl/fl^*/UUO mice and 9.08 ± 0.60% of *Hdac*3*^Fb−/−^*/AAI versus 19.43 ± 0.96% of *Hdac3^fl/fl^*/AAI mice, *P < 0.05; Fig. 2D and E) corroborated the Masson’s trichrome staining results. Consistent with reduced fibrotic alteration, protein expression analysis indicated that *Hdac3^fl/fl^* mice exhibited up-regulated α-SMA and Colla 1, along with marked suppression of RASAL1 after UUO or AAI treatment. In contrast, *Hdac*3*^Fb−/−^* mice showed milder alterations of these parameters (Fig. 2F and G). These findings demonstrate that HDAC3 is a critical upstream regulator of RASAL1 suppression and fibroblast activation, underscoring its pivotal role in driving FMT and the progression of renal fibrosis.

### HDAC3-selective inhibition alleviates RASAL1 suppression and renal fibrosis

**Fig. 3.**
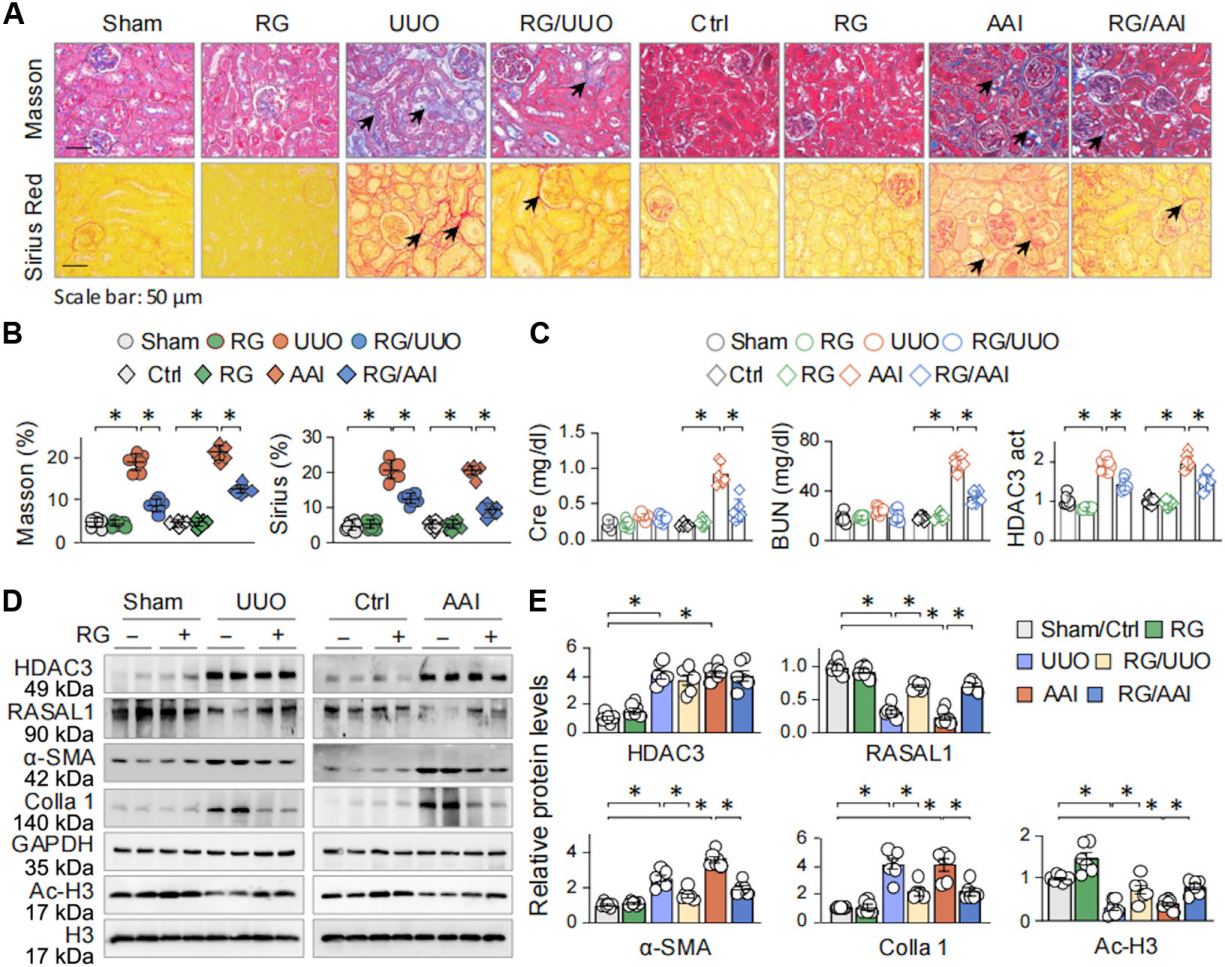
HDAC3 inhibition by RG mitigates RASAL1 suppression and fibrotic renal injuries of UUO and AAI mice. C57BL/6 mice were subjected to sham/UUO for 7 d or control/AAI for 14 d treated with or without RG (10 mg/kg; *n* = 6). (A) Representative photomicrographs of kidney sections from UUO (left half) and AAI (right half) mice stained by Masson’s trichrome (top) and Sirius Red (bottom). The collagen depositions are indicated by arrows. (B) Quantifications of collagen depositions based on (A). (C) Cre, BUN, and HDAC activity in renal tissues. (D) Western blots of HDAC3, RASAL1, α-SMA, Colla 1 and Ac-H3 in renal tissues. GAPDH and H3 served as the internal controls. Two randomly selected samples from each group were shown. (E) Quantifications of (D). Data were presented as means ± SEM. *P < 0.05, *n* = 6, 2-way ANOVA.

To further assess the functional significance of HDAC3 dysregulation in fibroblast activation, we used a pharmacological approach using RGFP966 (RG), a selective HDAC3 inhibitor with a median inhibitory concentration of 80 nM and negligible off-target effects on other HDAC isoforms up to 15 μM [29]. Mice were assigned to sham/control, RG, UUO or AAI, and RG-treated UUO (RG/UUO) or RG-treated AAI (RG/AAI) groups. RG administration had no detectable impact on normal kidney morphology but significantly attenuated fibrotic injuries induced by UUO (Masson, 8.79 ± 0.65% of RG/UUO versus 18.89 ± 0.86% of UUO mice; Sirius Red, 12.38 ± 0.59% of RG/UUO versus 20.61 ± 1.12% of UUO mice; *P < 0.05; Fig. 3A and B), and AAI treatment (Masson, 12.56 ± 0.42% of RG/AAI versus 21.22 ± 0.69% of AAI mice; Sirius Red, 9.28 ± 0.64% of RG/AAI versus 20.47 ± 0.60% of AAI mice; *P < 0.05; Fig. 3A and B). Serum creatinine (Cre) and blood urea nitrogen (BUN) levels remained in normal range in UUO mice, likely due to compensatory function of the contralateral kidney [30], but were elevated in AAI mice. RG treatment ameliorated this elevation (Fig. 3C), indicating systemic renal protection.

RT-PCR analysis revealed that RG reversed the suppression of *Rasal1* mRNA in fibrotic kidneys (Fig. S1C and D). Furthermore, RG corrected the altered expression of α-SMA, Colla 1, and RASAL1 (Fig. 3D and E). Mechanistically, RG reversed histone 3 (H3) hypoacetylation observed in UUO- or AAI-treated kidneys and restored acetylation at H3K4 and H3K18 (Fig. S1E and F), implicating these loci as HDAC3-sensitive epigenetic sites during fibrogenesis. Moreover, it significantly suppressed HDAC3 enzymatic activity enhanced by UUO and AAI (Fig. 3C, third graph). Together, these findings underscore the therapeutic potential of HDAC3 inhibition in sustaining RASAL1 expression, restraining FMT, and mitigating the progression of renal fibrosis.

### HDAC3 inhibits RASAL1 transcription in renal fibroblasts

**Fig. 4.**
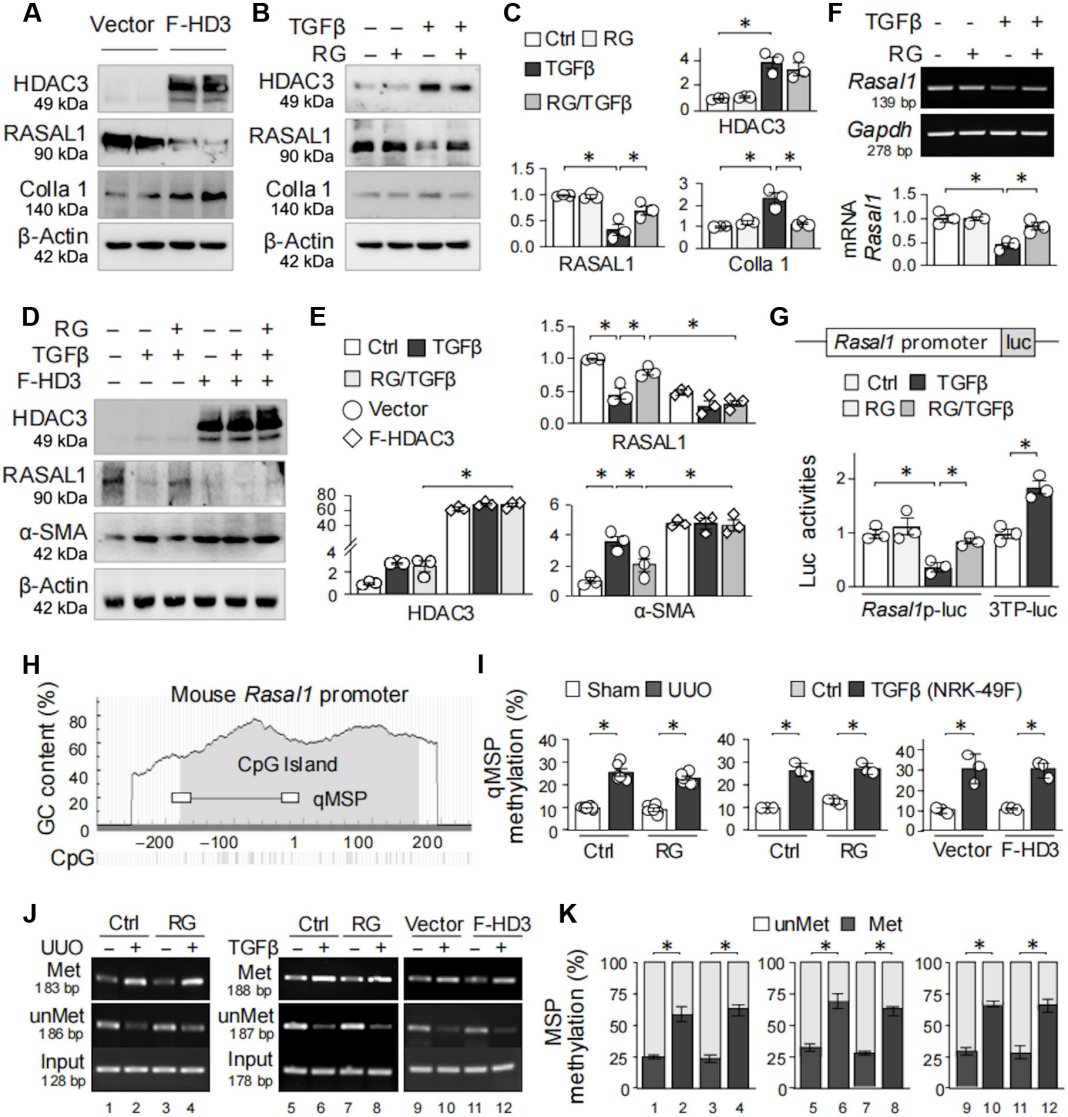
HDAC3 inhibits RASAL1 transcription in renal fibroblasts. (A) NRK-49F cells were transfected with either a control plasmid (vector) or a plasmid overexpressing Flag-tagged HDAC3 (F-HD3). Twenty-four hours later, the cell lysates were assayed for HDAC3, RASAL1, and Colla 1. β-Actin served as the internal control. (B) NRK-49F cells were treated with TGFβ (10 ng/ml) in the presence or absence of RG (10 μM) for 24 h, and then the cell lysates were tested for HDAC3, RASAL1, and Colla 1. (C) Quantitation of Western blots in (B). (D) Western blotting. NRK-49F cells transfected with vector or Flag-tagged HDAC3 plasmids for 24 h, followed by treatment with TGFβ (10 ng/ml) and/or RG (10 μM) for another 24 h. Cell lysates were assayed with antibody against HDAC3, RASAL1, and α-SMA. (E) Quantitation of (D). (F) NRK-49F cells were treated with TGFβ (10 ng/ml) in presence or absence of RG (10 μM) for 24 h, and the mRNA of *Rasal1* was analyzed by RT-PCR. *Gapdh* served as the internal control. The bottom panel is the quantitation. (G) Luciferase assay. HEK293T cells were transfected with either a positive control plasmid 3TP-luc or the *Rasal1* promoter reporter *Rasal1*p-luc together with a *Renilla* luciferase reporter and then treated with TGFβ (10 ng/ml) with or without RG (10 μM) for 24 h. Cell lysates were assayed for luciferase activities. (H) Schematic representation of the mouse *Rasal1* promoter region spanning −300 to +243 relative to the transcriptional start site. The CpG island (gray-shaded area) and positions of qMSP primers (boxed regions) are depicted. (I) qMSP analysis of the *Rasal1* promoter methylation in renal tissues from sham and UUO mice (7 d) (left) and in NRK-49F renal fibroblasts treated with TGFβ (10 ng/ml; 24 h) (right) with or without RG (10 mg/kg in mice; 10 μM in cell culture). (J) Representative agarose gel analysis of methylated (Met), unmethylated (unMet) and input PCR products for mouse tissue and NRK-49F cells mentioned above. (K) Quantitation of (J). Data were presented as means ± SD of 3 repeated cell assays or means ± SEM based on 6 renal samples. *P < 0.05, 2-tailed unpaired *t* test (3TP-luc) or 2-way ANOVA (*Rasal1*p-luc) (C, E, F, I, and K).

To determine whether HDAC3 directly regulates RASAL1 transcription, we assessed the impact of HDAC3 overexpression and inhibition on RASAL1 expression in renal fibroblasts. Transfection of NRK-49F cells with a plasmid encoding Flag-tagged HDAC3 resulted in suppressed RASAL1 levels and increased Colla 1 expression (Fig. 4A), mirroring the effects of TGFβ treatment, which was effectively corrected by RG treatment (Fig. 4B and C). Moreover, HDAC3 overexpression attenuated the protective effects of RG on α-SMA and RASAL1 expression (Fig. 4D and E). In NRK-49F cells, TGFβ-incurred *Rasal1* mRNA reduction was substantially relieved by RG (Fig. 4F). Furthermore, we constructed a mouse *Rasal1* promoter luciferase reporter plasmid, *Rasal1*p-luc, and found that TGFβ inhibited the *Rasal1*-promoter-driven luciferase activity, which was restored by RG (Fig. 4G). Notably, while increased *Rasal1* promoter methylation was confirmed in both UUO kidney and TGFβ-treated NRK-49F cells, methylation-specific PCR (MSP) and quantitative MSP (qMSP) analysis revealed that neither HDAC3 overexpression nor inhibition with RG altered methylation levels (Fig. 4H to K). These data indicate that HDAC3 directly suppresses RASAL1 transcription in renal fibroblasts independent of DNA methylation.

### HDAC3 transcriptional inhibition of RASAL1 is coregulated by YY1

**Fig. 5.**
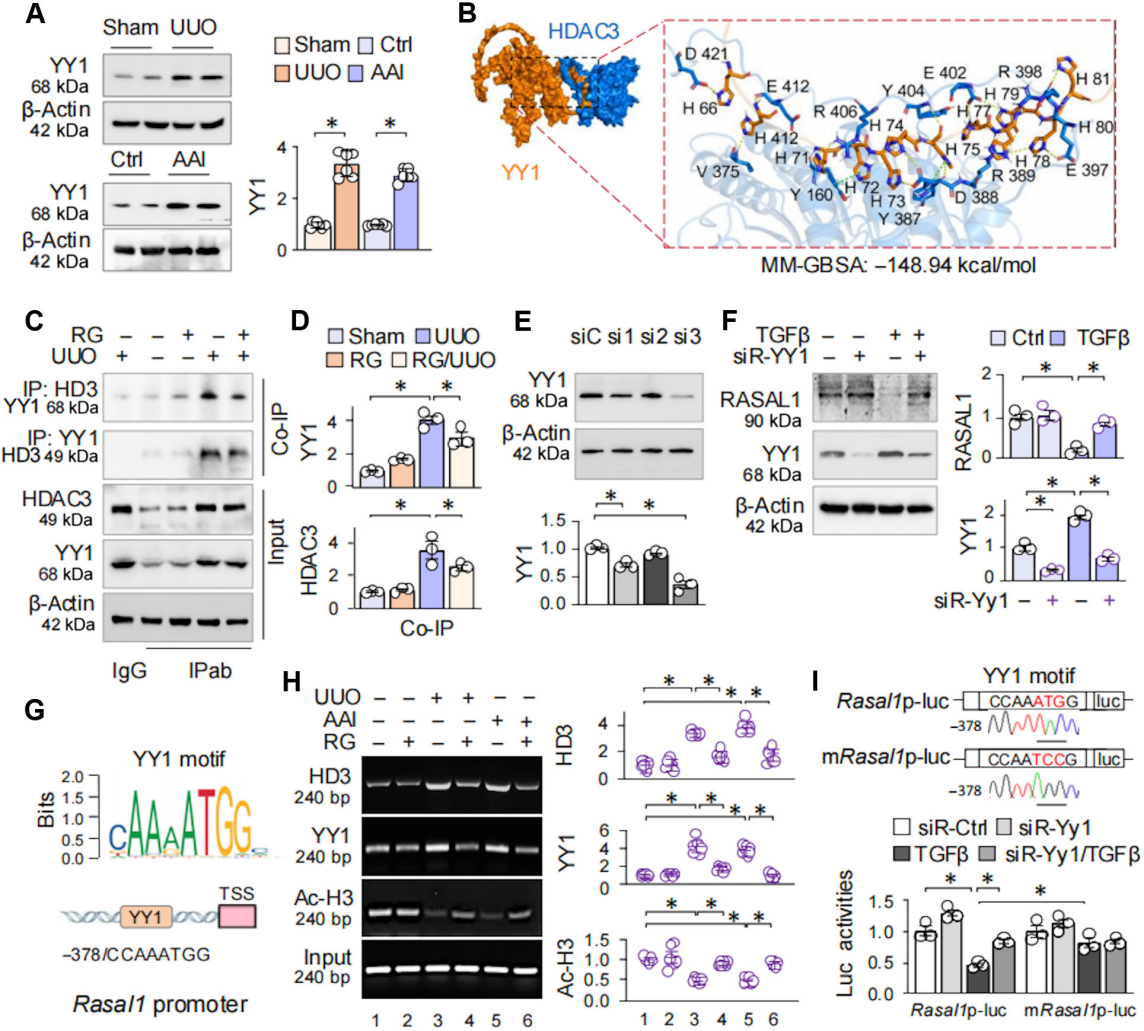
HDAC3 inhibition of *Rasal1* transcription involves YY1. (A) Western blots of renal tissues from sham-, UUO (7 d)-, control-, and AAI (14 d)-treated mice (*n*= 6) for YY1. The quantitation was on the right side. (B) Molecular docking between HDAC3 and YY1. The binding energy was analyzed by MM-GBSA. (C) Co-IP assay. Renal tissue homogenates from sham-, RG-, UUO-, or RG/UUO mice (*n*= 6) were assayed for HDAC3 and YY1 as input controls (the bottom 3 blots). Subsequently, the same tissue lysates were immunoprecipitated with isoform-matched immunoglobulin (Ig) or antibodies (IPab) specific to HDAC3 (HD3) or YY1, and then immunoprecipitants were reciprocally assessed for HDAC3 and YY1 by Western blotting (the top 2 blots). (D) Quantification of Co-IP in (C). Data were presented as means ± SEM based on 3 renal samples from each group. (E) NRK-49F cells were transfected with control lentivirus (siC) and 3 YY1 knockdown lentiviruses (si1, si2, and si3) for 48 h. Western blotting was performed on cell lysates to measure YY1 protein levels. The bottom panel was the quantification of protein. (F) NRK-49F cells were transfected with control lentivirus (siC) and YY1 knockdown lentivirus (siR-Yy1, si3) for 24 h, followed by treatment with TGFβ (10 ng/ml) for additional 24 h. Cell lysates were assayed for RASAL1 and YY1 expression by Western blotting. (G) The top panel shows the YY1 binding motif sequence logo from JASPAR, while the bottom panel depicts a schematic diagram of YY1 binding to the *Rasal1* promoter. TSS, transcription start site. (H) ChIP assay. The control, UUO, or AAI mice were treated with or without RG (10 mg/kg). The renal tissues indicated above were immunoprecipitated with antibodies to HDAC3, YY1 and Ac-H3, and then the precipitated genomic DNA (input) and the antibody-bound DNAs were PCR amplified with primers covering the YY1 motif on *Rasal1* promoter. The RT-PCR products were analyzed on an agarose gel (left). RT-qPCR (right) was normalized to input DNA and presented as fold changes relative to sham. (I) Luciferase assay. HEK293T cells were transfected with the murine *Rasal1* promoter reporter (*Rasal1*p-luc) or the m*Rasal1*p-luc plus a *Renilla* luciferase plasmid. After 24 h of YY1 knockdown and control lentivirus treatment, cells were treated with TGFβ (10 ng/ml) for additional 24 h, and the luciferase activities were measured and normalized to *Renilla* luciferase activities. Data were presented as means ± SEM based on 6 renal samples or means ± SD of 3 repeated cell assays. *P < 0.05, one-way ANOVA (A and E), 2-way ANOVA (D, F, and H), or 3-way ANOVA (I).

HDAC3 is known to repress transcription exclusively by forming complexes with other transcriptional regulators that guide it to specific gene promoters [25,31]. To dissect the mechanism underlying HDAC3-mediated RASAL1 transcriptional inhibition, we examined the *Rasal1* promoter using JASPAR and identified several binding motifs for transcription factors related to renal fibrosis, including NF-κB1 (−1,088/gggtcatccc, binding score 8.5559), FOXO3 (−1,722/aaataaacacac, 10.7895), and nuclear factor erythroid 2-related factor 2 (−1,543/gagcatgacacgaca, 9.8981) [32–34]. However, a YY1 binding motif near the transcription start site exhibited a very high binding score (−378/taccaaatggca, 10.4597). Given YY1’s established role in fibroblasts [35–38] and its known involvement in HDAC3-associated transcriptional suppression [39], we hypothesized that YY1 recruits HDAC3 to repress RASAL1 transcription. YY1 expression was found to be elevated in UUO- and AAI-treated kidneys (Fig. 5A). Molecular docking predicted a strong interaction between HDAC3 and YY1, with a molecular mechanics/generalized born surface area (MM-GBSA) score of −148.94 kcal/mol (Fig. 5B), suggesting a strong interaction between these 2 proteins. Moreover, coimmunoprecipitation (co-IP) confirmed inducible association of HDAC3 with elevated YY1 in UUO kidneys, and this association was not disrupted by RG treatment (Fig. 5C and D).

To functionally interrogate YY1’s role, we generated 3 YY1 knockdown lentiviruses (siR-Yy1-1, siR-Yy1-2 and siR-Yy1-3), identifying siR-Yy1-3 as the most effective (Fig. 5E and Fig. S1G). YY1 knockdown mitigated TGFβ-induced RASAL1 suppression in fibroblasts (Fig. 5F). Chromatin immunoprecipitation (ChIP) assays targeting the YY1 binding site on the *Rasal1* promoter (*−*378/TACCAAATGGCA) revealed enrichment of HDAC3 and YY1 at this locus in UUO- and AAI-treated kidneys, accompanied by local hypoacetylation, which was reversed by RG treatment (Fig. 5G and H). To further validate YY1’s functional role, we constructed a mutant *Rasal1*p-luc reporter (m*Rasal1*p-luc), in which ATG in the YY1 motif was replaced by TCC. Upon TGFβ stimulation, luciferase activity was markedly reduced in the wild-type reporter but remained largely unaffected in the mutant construct. YY1 knockdown restored luciferase activity of *Rasal1*p-luc but not that of the mutant reporter (Fig. 5I). Collectively, these findings demonstrate that HDAC3 collaborates with YY1 to suppress *Rasal1* transcription under renal fibrotic conditions, potentially via recruitment to a high-affinity YY1 motif and local histone deacetylation.

### RASAL1 derepression is essential for the antifibrosis role of HDAC3 inhibition in vitro and in vivo

**Fig. 6.**
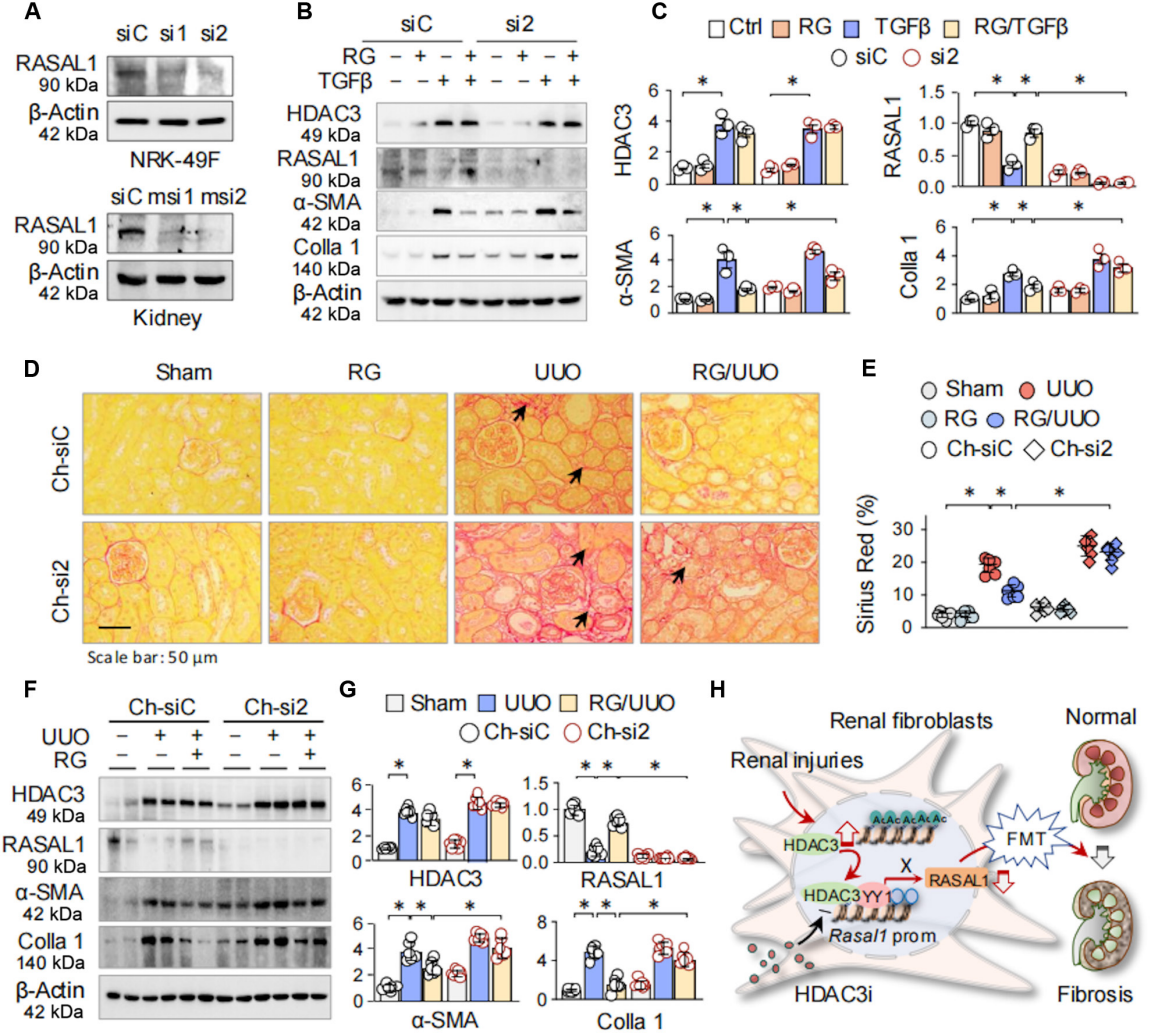
RASAL1 derepression is essential for the antifibrosis function of HDAC3 inhibition in vitro and in vivo*.* (A) Top: NRK-49F cells were treated with siC, si1, or si2 for 48 h. Western blotting of the cell lysates was assayed for RASAL1 proteins. Bottom: C57BL/6 mice were treated with Ch-siC, Ch-si1, or Ch-si2 once by intravenous injection, 6 mice in each group. Seven days later, Western blotting was performed on mouse renal tissues to detect RASAL1 proteins. (B) Western blotting. NRK-49F cells were infected with siC or si2 for 48 h, and then the cells were treated with TGFβ (10 ng/ml) and/or RG (10 μM) for 24 h. Cell lysates were assayed with antibody against HDAC3, RASAL1, α-SMA, and Colla 1. (C) Quantifications of Western blots in (B). Data were presented as means ± SD of 3 repeated cell assays. *P < 0.05, 3-way ANOVA followed by Tukey’s post hoc test. (D) Mice receiving Ch-siC or Ch-si2 were subgrouped into sham, RG, UUO, or RG/UUO (*n* = 6). Representative photomicrographs of kidney sections were stained by Sirius Red. The black arrows indicate fibrotic areas. (E) Quantification of (D). Data were presented as scatter plot. *P < 0.05, 3-way ANOVA followed by Tukey’s post hoc test. (F) Western blotting. The renal tissues were assayed for HDAC3, RASAL1, α-SMA, and Colla 1. Two samples from each group were shown. (G) Quantifications of (F). Data were presented as means ± SEM based on 6 renal samples. *P < 0.05, 2-way ANOVA. (H) A schematic diagram of sequential HDAC3 elevation, RASAL1 suppression, and FMT during renal fibrosis. Elevated HDAC3, in association with YY1, induces hypoacetylation and transcriptional suppression of the *Rasal1* promoter, resulting in persistent FMT and development of renal fibrosis (red lines). Conversely, selective HDAC3 inhibition (HDAC3i) by RG mitigates *Rasal1* suppression (black lines), FMT, and renal fibrosis.

To elucidate the critical role of HDAC3-inhibition-mediated RASAL1 derepression in FMT and renal fibrosis, we designed small interfering RNAs (siRNAs) targeting Rasal1 in NRK-49F cells, namely, siR-Rasal1-1 (si1) and siR-Rasal1-2 (si2), and cholesterol-conjugated siRNAs (Ch-siRNAs) for in vivo Rasal1 silencing in mouse kidneys, namely, Ch-siR-Rasal1-1 (Ch-si1) and Ch-siR-Rasal1-2 (Ch-si2). RT-PCR (Fig. S1H and I) and Western blotting (Fig. 6A) revealed that si2 and Ch-si2 achieved more effective knockdown than their respective counterparts and were selected for subsequent experiments.

In vitro, NRK-49F cells transfected with either control siRNA (siC) or si2 were treated with TGFβ in the presence or absence of RG. RASAL1 protein levels were markedly reduced in si2-transfected cells, while HDAC3 expression remained unaffected. Notably, RG normalized TGFβ-induced up-regulation of α-SMA and Colla 1 in siC cells but failed to do so in si2-transfected cells (Fig. 6B and C), indicating that RASAL1 restoration is indispensable for the anti-FMT effects of HDAC3 inhibition in fibroblasts.

In vivo, mice injected with Ch-siC or Ch-si2 were subjected to sham, RG, UUO, or RG/UUO treatment. Consistent with in vitro results, Ch-si2-treated mice displayed significant reduction in RASAL1 expression, which correlated with exacerbated fibrotic changes under both basal and UUO conditions. RG markedly attenuated fibrosis in Ch-siC mice (Sirius Red, 11.51 ± 0.80% of RG/UUO versus 19.50 ± 0.94% of UUO, *P < 0.05), but this protective effect was largely abolished in Ch-si2-treated mice (Fig. 6D and E and Fig. S1J and K). Similarly, RG normalized UUO-induced aberrant expression of α-SMA and Colla 1 in Ch-siC mice, but this effect was significantly blunted in Ch-si2-injected mice (Fig. 6F and G). Collectively, these findings underscore the necessity of RASAL1 restoration for the renoprotective effects of RG in experimental renal fibrosis.

## Discussion

In this study, we have made informative findings to better understand the epigenetic mechanism underlying RASAL1 suppression in the pathogenesis of renal fibrosis. We demonstrate that HDAC3 elevation, in concert with the transcriptional coregulator YY1, represses RASAL1 transcription and promotes FMT in UUO- and AAI-induced fibrotic kidneys. Notably, both genetic ablation and pharmacological inhibition of HDAC3 using RG restore RASAL1 levels and effectively attenuate FMT and renal fibrotic pathology (Fig. 6H). These findings unveil a previously uncharacterized HDAC3–YY1–RASAL1 axis as a central epigenetic driver of FMT in renal fibrosis and underscore its potential as a promising target for therapeutic intervention.

The identification of HDAC3 as a fibroblast-specific epigenetic regulator of RASAL1 adds depth to our understanding of FMT programming in renal fibrosis, a process widely considered irreversible once myofibroblasts emerge [40]. FMT is intricately regulated by complex signaling networks, including TGFβ, Wnt/β-catenin, and NF-κB, which operate across diverse cell types and involve various HDAC isoforms [41]. Epigenetic imprinting of fibrotic genes and sustained myofibroblast proliferation are key contributors to the persistence of myofibroblasts in fibrotic tissues [42,43]. Several HDACs have been implicated in renal fibrosis. HDAC1, HDAC6, and HDAC9, for example, promote renal fibrosis by modulating fibrotic and inflammatory signaling pathways in tubular epithelial cells [44–46], while HDAC4, despite possessing limited intrinsic deacetylase activity, promotes renal fibrosis primarily through its scaffolding functions [47]. In contrast, our findings establish a unique fibroblast-specific function for HDAC3: It represses the antifibrotic gene RASAL1 via direct interaction with the transcription factor YY1 and promotes FMT. This distinct regulatory axis sets HDAC3 apart from other isoforms and underscores its central function in driving fibroblast reprogramming and FMT, positioning HDAC3 as a selective and promising therapeutic target for halting renal fibrotic diseases.

Beyond epigenetic reprogramming effects in fibroblasts, HDAC3 inhibition may attenuate renal fibrosis through additional mechanisms involving tubular epithelial and immune cells. In animal models of renal fibrosis, pharmacological blockade of HDAC3 restores expression of the epithelium-specific antifibrotic factor Klotho [20], suppresses the necroptosis initiator receptor-interacting protein kinase 1 [48], and NF-κB-dependently promotes macrophage polarization toward an anti-inflammatory M2 phenotype [49]. The present study extends these findings by revealing a direct and previously unrecognized role of HDAC3 in renal fibroblasts, where it epigenetically represses RASAL1, driving myofibroblast activation and extracellular matrix production—the ultimate effector mechanism of fibrosis. Thus, HDAC3 inhibition by RG exerts complementary protective effects at distinct stages and in different renal cell populations: (a) preserving tubular epithelial cell survival (antiferroptosis), (b) dampening macrophage-mediated inflammation, and (c) directly restraining fibroblast-to-myofibroblast transformation and matrix deposition. Collectively, these ancillary actions complement the direct epigenetic regulation of RASAL1 by HDAC3 inhibition in fibroblasts and synergistically enhance its antifibrotic efficacy.

RASAL1 promoter methylation is a well-established mechanism of transcriptional silencing in renal fibrosis [12]. DNA-methylation-mediated gene transcriptional repression is typically orchestrated by a multicomponent repressive complex comprising DNMTs, methylcytosine-binding domain proteins (which harbor transcriptional repressor domains that recruit corepressors), and HDACs [50,51]. The coordinated interplay between DNA methylation and histone modifications is a central feature of epigenetic regulation [15]. This functional connection is further supported by evidence that DNMT1 is associated with HDAC activities [52], although its role in RASAL1 suppression during renal fibrosis remains undefined. In this study, we confirm that the Rasal1 promoter is heavily methylated. However, modulation of HDAC3—either by pharmacological inhibition or overexpression—does not affect *Rasal1* promoter methylation in fibrotic kidneys or renal fibroblasts (Fig. 4H to K). These findings support a cooperative epigenetic regulatory mode in which HDAC3 controls RASAL1 transcription through histone deacetylation, functioning in conjunction with DNA-methylation-mediated repression.

A particularly novel and mechanistically informative discovery in this study is the identification of YY1 as a functional partner of HDAC3 in repressing RASAL1 transcription. YY1 is a ubiquitously expressed transcription factor belonging to the GLI-Kruppel class of zinc finger proteins, with the capacity to either activate or repress gene transcription, depending on its interacting partners, promoter context, and chromatin architecture [53]. YY1 has been implicated in the regulation of cell proliferation, migration, and invasion across various cancers [54,55]. Previous studies have shown that YY1 and HDAC3 co-occupy the YY1 motif within the c-Myc promoter, leading to chromatin deacetylation and transcriptional silencing of c-Myc [39]. In addition, YY1 bound to the pleckstrin homology-like domain family A member 2 (PHLDA2) promoter and recruited HDAC1 and HDAC3 to corepress PHLDA2 transcription [56]. The direct interaction between YY1, protein arginine methyltransferase 7, and HDAC3 has been reported to suppress E-cadherin expression, thereby attenuating epithelial-to-mesenchymal transition and metastasis in breast cancer [57]. Despite these insights, the role of YY1 in FMT and renal fibrosis has remained undefined. In this study, we demonstrate that YY1 expression is elevated in fibrotic kidneys induced by UUO and AAI. We further show that YY1 physically interacts with HDAC3 at the *Rasal1* promoter, leading to *Rasal1* transcriptional suppression. These findings establish YY1 as a previously unrecognized mediator of HDAC3-driven RASAL1 suppression, with potential implications for antifibrotic therapy.

In conclusion, our findings demonstrate that aberrant HDAC3 elevation, together with YY1, represses *Rasal1* transcription and promotes FMT in the fibrotic kidneys incurred by UUO and AAI. Pharmacological inhibition of HDAC3 with RG restores RASAL1 expression and effectively mitigates FMT and renal fibrosis. Given that FMT is a pathological hallmark shared across multiple organs [58], the HDAC3–YY1–RASAL1 axis identified in this study may represent a conserved epigenetic mechanism underlying systemic fibrotic disorders. These findings highlight HDAC3 and its cofactor YY1 as promising pan-fibrotic therapeutic targets. Further investigation may determine whether this mechanistic pathway contributes more broadly to other fibroblast-driven processes, such as extrarenal fibrosis, tumor-metastasis-associated desmoplasia, dysregulated wound healing, and tissue regeneration.

## Materials and Methods

### Animal studies

C57BL/6 mice were purchased from JunKe Biological, Nanjing, China. To generate the conditional *Hdac3^Fb−/−^*, *Hdac3^fl/fl^* mice containing 2 loxP sites, one upstream of exon 11 and the other downstream of exon 14 of *Hdac3* gene [20], were bred with transgenic *Col1a2*-CreER mice (C001248, Cyagen Biosciences, Suzhou, China). Mouse genotype was confirmed by PCR with mouse tail DNA using the following primers: F1, CAGGAGGTTTCGACTAAGTTGG; R1, CATGTCCATCAGGTTCTTGC; F2, GATTGGTCTCGTCTGGCGCT; R2, GGCTAGTGGGCGCATGTA; F3, GCTTGGTAGCCAGCCAGCTTAG; and R3, CATGTGACCCCAGACATGACTGG. To induce *Hdac3* deletion, *Hdac3^Fb−/−^* mice were injected intraperitoneally with tamoxifen (1 mg per mouse, dissolved in corn oil containing 10% ethanol) for 5 consecutive days and waited for 7 additional days before experiments.

Two mouse models of renal fibrosis were established with 8-week-old male mice, namely, UUO (the left ureter was exposed via a flank incision, ligated with 4-0 silk at 2 points, and cut between the 2 ligation, and mice were then maintained for 7 d) and AAI (A5512, Sigma-Aldrich, USA; 5 mg/kg, intraperitoneal injection every other day for 14 d) [20]. For study to determine the phenotype of *Hdac3* knockout, *Hdac3^fl/fl^* and *Hdac3^Fb−/−^* mice were injected with tamoxifen first as described above and then subjected to UUO and AAI. For intervention study, C57BL/6 mice were randomly subjected to 4 groups: (a) sham/vehicle control, (b) RG treatment (HY-13909, MedChemExpress, USA; 10 mg/kg, subcutaneous injection starting the first day and following every other day) [59], (c) renal fibrosis model mice: UUO for 7 d or AAI for 14 d, and (d) RG intervention in UUO/AAI mice. For study to determine the role of RASAL1 in HDAC3 intervention, C57BL/6 mice were injected through tail vein with either a Ch-siRNA control containing a scrambled sequence or a Ch-siRNA targeting RASAL1 and then subjected to control, RG, UUO, or RG/UUO. The mice were euthanized by spinal subluxation after the experiment completion, and then the mouse kidneys were surgically collected, fixed for histological assay, or stored at −80 °C for further analysis.

Mice were housed under 25 ± 2 °C temperature, 50% to 60% humidity, and a 12-h light/dark cycle conditions with ad libitum access to food and water. For all animal experiments, 6 mice per group (*n* = 6) were used. Mice were randomly assigned to experimental groups, and investigators were blinded to group allocation during outcome assessment. No animals were excluded from experiments unless technical failure occurred.

### Human kidney samples

Kidney biopsies from 16 patients were obtained from the Yancheng Biobank at The First People’s Hospital of Yancheng, Yancheng, China. Patients were classified 2 groups based on CKD stage and the degree of tubulointerstitial fibrosis. Mild kidney injury group (*n* = 6) consisted of patients with CKD at stages 1 to 2 with mildly impaired renal function and ≤10% tubulointerstitial fibrosis. Severe CKD group (*n* = 10) included these at CKD stage ≥3 with >10% tubulointerstitial fibrosis. Detailed patient information is provided in Table S1.

### Cre and BUN assay

Renal function in the experimental mice was assessed by anesthetizing them and collecting blood from the orbital sinus with a capillary glass tube. The blood samples were centrifuged at 4,000 rpm for 20 min at 4 °C to obtain serum. Cre and BUN levels (in milligrams per deciliter) were measured using the creatinine assay kit (D799853, Sangon Biotech, China) and the urea nitrogen assay kit (BC1535, Solarbio, China), respectively.

### Masson’s trichrome and Sirius Red staining

Kidney tissues were paraffin embedded, cut into 4-μm sections, and stained using Masson’s trichrome or Sirius Red staining as before [20]. To evaluate the fibrosis extent, the sections were photographed with an Olympus BX53 light microscope and blindly evaluated using ImageJ software. Ten randomly selected, nonoverlapping areas were assessed per section, and the average proportion of positively stained field to the whole examined area was calculated.

### IHC staining

IHC staining of kidney sections was conducted following previously established protocols [20] with primary antibodies to HDAC3 (A2139, RRID: AB_2764158, Abclonal, China) and RASAL1 (PA5-95580, RRID: AB_2807382, Thermo Fisher Scientific, USA) and examined with an Olympus BX53 microscope. Average optical density was quantified using Image-Pro Plus 6.0 software (Media Cybernetics Inc., USA) to analyze the semiquantitative expression of HDAC3 and RASAL1 [60]. Five randomly selected fields in each section were evaluated, and all assessments were performed in a double-blind manner.

### HDAC3 activity assay

HDAC3 activities of kidney tissues were assessed using an HDAC activity/inhibition direct assay kit (colorimetric) (P-4034, Epigentek, USA). Following the manufacturer’s instructions, the acetylated substrates were incubated with the renal tissue homogenates with or without the HDAC3 inhibitor RG at 37 °C for 60 min. Active HDACs deacetylate the substrates, and the level of deacetylated products reflects enzyme activity. Absorbance at 450 nm was measured using a multimode microplate reader (Varioskan LUX, USA) after adding the color developer solution. HDAC activities were expressed as fold changes compared to the control, and the decreased activities induced by RG were considered the HDAC3 activity [18].

### Isolation and culture of mouse primary renal fibroblasts

Primary renal fibroblasts from mice were isolated and cultured following previously established protocols [61]. Briefly, kidneys from 8-week-old male mice were minced into small fragments and digested in phosphate-buffered saline (PBS) containing collagenase II (17101015, Thermo Fisher Scientific, USA) at 37 °C for 45 min. The resulting cell suspension was filtered through a 70-μm mesh and centrifuged at 1,000*g* for 5 min. The pellet was washed with PBS, treated with erythrocyte lysis buffer for 3 min, and then centrifuged again at 1,000*g* for 5 min to remove the supernatant. Finally, the cells were resuspended in Dulbecco’s modified Eagle’s medium/Ham’s F-12 (DMEM/F12) medium supplemented with 10% fetal bovine serum and 1% penicillin/streptomycin for culture.

### Cell culture and treatment

Human embryonic kidney (HEK) 293T cells (American Type Culture Collection, USA) were cultured in DMEM, and rat renal fibroblast NRK-49F cells (Procell Life Science & Technology, Wuhan, China) and mouse primary renal fibroblasts were cultured in DMEM/F12 medium. Both media were supplemented with 10% fetal bovine serum and 1% penicillin/streptomycin (Gibco, USA) in a humidified atmosphere of 5% CO_2_ incubator at 37 °C. Cells were treated with TGFβ (240-B-002, R&D Systems, USA), AAI, and the HDAC3-selective inhibitor RG as indicated.

### RASAL1 knockdown by RNA interferences

For in vitro RASAL1 knockdown from NRK-49F cells, 2 siRNAs (RsiRNA-*Rasal1*-1, 5′-AACGTGAATGACCTCAATCAA-3′; RsiRNA-*Rasal1*-2, 5′-CACCCGCTTTGCCTTCAAGA-3′) targeting rat RASAL1 mRNA were tested (GenScript, Nanjing, China). The siC contained a scrambled RNA sequence (5′-TTCTCCGAACGTGTCACGT-3′). The siRNAs at 50 nM were transfected into NRK-49F cells by Lipofectamine 3000 (Invitrogen, USA). Twelve hours after transfection, the cells were treated with RG and/or TGFβ as indicated. Twenty-four hours later, the cell lysates were examined by Western blotting.

For in vivo RASAL1 knockdown, 2 Ch-siRNAs targeting mouse RASAL1 (Ch-siRNA-*Rasal1*-1, 5′-AACGTGAATGACCTCAACCAA-3′; Ch-siRNA-*Rasal1*-2, 5′-CACGCGCTTTGCCTTCAAGAA-3′) [12] were commercially synthesized by GenScript (Nanjing, China). A scrambled cholesterol-conjugated siRNA (5′-TTCTCCGAACGTGTCACGT-3′) was used as a negative control. Each siRNA was administered as a single tail-vein injection at a dose of 10mg/kg in 200 μlofPBS per mouse [62,63].

### Lentivirus-mediated YY1 knockdown

YY1 knockdown and control lentivirus were constructed by GeneChem (Shanghai, China). Three lentiviruses targeting the following YY1 mRNA of NRK-49F cells: siRNA-Yy1-1 (5′-GGGTAATAAGAAGTGGGAACA-3′), siRNA-Yy1-2 (5′-GCGAGTTCTCGGTCACCATGT-3′), and siRNA-Yy1-3 (5′-GCGTTCGTTGAGAGCTCAAAG-3′). The control lentivirus carried a scrambled RNA sequence (5′-TTCTCCGAACGTGTCACGT-3′). The lentivirus at a dose of 10 multiplicities of infection (MOIs) (2 × 10^9^ transducing units [TU]/ml) was transduced into NRK-49F cells by HiTranG A infection enhancement reagents. After 24 h, transfected cells were treated with TGFβ as indicated. Cell lysates were examined 24 h later for Western blotting in subsequent experiments.

### Western blotting

Cells and kidney tissues were lysed in radioimmunoprecipitation assay lysis buffer (P0013B, Beyotime, China) supplemented with protease inhibitor cocktail (P1005, Beyotime, Shanghai, China), followed by sonication. Protein concentration was measured with bicinchoninic acid assay kit (23225, Thermo Fisher Scientific, USA), and the lysates were analyzed by SDS–polyacrylamide gel electrophoresis. The primary and secondary antibodies used were as follows: HDAC3 (A2139, RRID: AB_2764158), α-SMA (A17910, RRID: AB_2861755), Colla 1 (A1352, RRID: AB_2760381), YY1 (A19569, RRID: AB_2862674), H3 (A2348, RRID:AB_2631273), glyceraldehyde-3-phosphate dehydrogenase (GAPDH; AC033, RRID: AB_2769570), and β-actin (AC026, RRID: AB_2768234) of ABclonal, Wuhan, China; RASAL1 (PA5-95580, RRID: AB_2807382); acetylated H3 (Ac-H3, 39139, RRID: AB_2687871, Active Motif, CA, USA); acetyl-H3 (Lys^4^) (H3K4ac, PTM-188, RRID: AB_3678562) and acetyl-H3 (Lys^18^) (H3K18ac, PTM-158, RRID: AB_3678563) from PTM BIO, Hangzhou, China; goat anti-rabbit immunoglobulin G (IgG)-horseradish peroxidase (HRP) (E-AB-1003, RRID: AB_2921220), and goat anti-mouse IgG-HRP (E-AB-1001, RRID: AB_2715613) from Elabscience Biotech, Wuhan, China. The blots were detected using an enhanced chemiluminescence plus Western blotting system (Vazyme, Nanjing, China), and the protein expression levels were quantified with ImageJ software after normalization to loading controls.

### Reverse transcription polymerase chain reaction

Total RNAs was extracted from mouse kidneys or cells using a total RNA extraction kit (R701-01, Vazyme). Equal amounts of mRNA were then reverse-transcribed into cDNA using the HiScript RT SuperMix Kit (R223-01, Vazyme). The cDNAs were analyzed by regular PCR with the following primers: mouse *Rasal1* (forward, ATCAAGAAGACCCGCTTTCCA; reverse, GAACTCCACCATACCCAGGAA), mouse *Gapdh* (forward, AACGACCCCTTCATTGAC; reverse, TCCACGACATACTCAGCA); rat *Rasal*1 (forward, 5′-CATCAAGAAGACCCGATTCCCA-3′; reverse, 5′- GAATTCCACCATGCCCAGGAA-3′), rat *Yy1* (forward, 5′-GAAAGCATCTGCACACCCAC-3′; reverse 5′-CAGCCTTCGAATGTGCACTG-3′), rat *Gapdh* (forward, 5′-GATTTGGCCGTATCGGAC-3′; reverse, 5′-GAAGACGCCAGTAGACTC-3′). The PCR products were analyzed and visualized on a 1.5% agarose gel.

### Plasmid construction, transfection, and luciferase assay

The positive control plasmid containing 3 copies of TGFβ/Smad-responsive element 3TP-luc has been previously described [19]. Mouse *Rasal1* promoter reporter plasmid *Rasal1*p-luc, encompassing 2,000 base pairs of the mouse proximal promoter region, was generated by amplifying genomic DNA from mouse tissue using PCR with forward primer CCGCTCGAGTCTTTCTGGCTCAGCCTCCTGTTC and reverse primer CCCAAGCTTGTCTCCAATTAAACCCGGAGTGTGC and cloned in pGL3-basic vector (Promega, USA) at the Xho I and Hind III sites (the cloning sites were underlined). A mutant m*Rasal1*p-luc was generated by PCR-based mutagenesis, in which the YY1 motif TACCAAATGGCA was mutated to TACCAATCCGCA and confirmed by sequencing. A *Renilla* luciferase reporter plasmid was cotransfected as an internal control to normalize the luciferase assay results. Cells were transiently transfected with 3TP-luc, *Rasal1*p-luc, or m*Rasal1*p-luc by Lipofectamine 3000. After 12 h of transfection, some cells were transduced with control or YY1-targeting lentivirus at a dose of 10 MOIs (2 × 10^9^ TU/ml) and then treated with HDAC3-selective inhibitor RG and/or TGFβ as indicated. Twenty-four hours later, the cell lysates were examined for luciferase activities with a dual luciferase reporter assay system (E1910, Promega, USA). The luciferase activities of the *Rasal1* promoter reporter were normalized to *Renilla*’s and reported as relative fold changes.

### HDAC3 overexpression assays

A plasmid overexpressing Flag-tagged HDAC3 [20] or a control vector was transfected into NRK-49F cells by Lipofectamine 3000. Twelve hours after transfection, the transfected cells were treated with RG and/or TGFβ as indicated. Cell lysates were examined by Western blotting 24 h later.

### Molecular docking

Because of the lack of resolved crystal structures for HDAC3 and YY1 proteins, we used predicted 3-dimensional structures generated by AlphaFold2. The protein models underwent a series of preprocessing steps, including the addition of hydrogen atoms, optimization of hydrogen bonding networks, assignment of bond orders, processing of disulfide bridges, and energy minimization to ensure structural stability. Protein–protein docking was subsequently carried out using the Piper module in Schrödinger software suite. A standard docking protocol was applied, with 70,000 rotatable ligand probes set as parameters. The top-ranked conformation from the output ensemble was selected as the final docking pose for further analysis.

### Coimmunoprecipitation

Co-IP was performed to detect the reciprocal protein associations as previously described [64]. The kidney samples were lysed in lysis buffer for Western blotting and IP (P0013, Beyotime, Shanghai, China) and incubated with antibodies against HDAC3 (A2139, RRID: AB_2764158, ABclonal, China), YY1 (#46395, RRID: AB_2799302, Cell Signaling Technology, USA), or isoform-matched immunoglobulin (IgG, AC005, RRID: AB_2771930, ABclonal, China), and then the immunoprecipitants were subjected to Western blotting with primary antibody against HDAC3 or YY1, respectively, and secondary antibody: HRP-conjugated mouse anti-rabbit IgG light chain (AS061, RRID: AB_2864055, ABclonal, China).

### Chromatin immunoprecipitation

ChIP assays were carried out using a ChIP assay kit (P2078, Beyotime, Shanghai, China). Three antibodies anti-HDAC3 (A2139, RRID: AB_2764158, ABclonal, China), anti-YY1 (#46395, RRID: AB_2799302, Cell Signaling Technology, USA) and anti-Ac-H3 (39139, RRID: AB_2687871, Active Motif, CA, USA) were used in the IP step, respectively. The input DNA and immunoprecipitated DNA were analyzed by regular PCR using the primer set YY1bF: TGTCATGCTCCCTGAACAGATG and YY1bR: CATGGGAGCCCTCTATCTATGG (covering −467/−228 region), which cover the YY1 binding motif on Rasal1 promoter (TACCAAATGGCA, −378 to −367). The PCR products were analyzed by RT-PCR and further quantified by quantitative real-time PCR (RT-qPCR). The RT-PCR products were analyzed by 1.5% agarose gel and quantitated by ImageJ software. The RT-qPCR was performed by the QuantStudio 5 Real-Time PCR System (Applied Biosystems, USA) and quantitated by 2^−ΔΔCt^ method.

### MSP and qMSP

CpG island prediction and MSP/qMSP primer design for the *Rasal1* promoter were performed using MethPrimer. Genomic DNA was extracted using the TIANamp Genomic DNA Kit (DP304, TIANGEN, China), followed by bisulfite conversion with the EpiArt Ultrafast Magnetic DNA Methylation Bisulfite Kit (EM113, Enzyme, China) according to the manufacturer’s instructions. Bisulfite-converted DNA was used to assess *Rasal1* promoter methylation by MSP/qMSP. The following primer sets were used for mouse kidney tissues: methylated primers mRAL-MF: AATTTTTATGAAAGGTTTAAGTCGT and mRAL-MR: AAACCCGAAATATACCAACGAA (−193/−11 locus), unmethylated primers mRAL-unMF: AATTTTTATGAAAGGTTTAAGTTGT and mRAL-unMR: ATTAAACCCAAAATATACCAACAAA (−193/−8 locus), and input DNA control primers Inp-mRALF: AGATGGCTCTTATCGTGCCG and Inp-mRALR: TTAAACCCGGAGTGTGCCAG (−136/−9 locus). The *Rasal1* promoter methylation of NRK-49F cells was assayed with methylated primers rRAL-MF: TAATTTTTATGAAAGGTTTAAGCGG and rRAL-MR: AATTAAACCCGAAATATACCAACG (−194/−7 locus), unmethylated primers rRAL-unMF: TAATTTTTATGAAAGGTTTAAGTGG and rRAL-unMR: ATTAAACCCAAAATATACCAACAAA (−194/−8 locus), and input DNA control primers Inp-rRALF: CATGAAAGGCTCAAGCGGTG and Inp-rRALR: TTAAACCCGGAGTGTGCCAG (−187/−9 locus). MSP products were analyzed on a 1% agarose gel and quantified using ImageJ software. The qMSP was performed by the QuantStudio 5 Real-Time PCR System (Applied Biosystems, USA) .

### Statistics

Statistical analysis was performed with GraphPad Prism 9.0. Data were presented as means ± SEM of animal experiments or means ± SD of cell experiments. Two-tailed unpaired *t* test or one-way analysis of variance (ANOVA) was used to assess differences between 2 groups. For comparisons of multiple groups, 2-way ANOVA or 3-way ANOVA followed by Tukey’s post hoc test was applied. Data normality and homogeneity of variances were assessed using the Shapiro–Wilk test and Levene’s test, respectively. A *P* value of less than 0.05 was considered statistically significant.

## Ethical Approval

The study protocol for utilizing the patient’s renal samples was approved by the ethics committee of The First People’s Hospital of Yancheng (project number: 2022-K-101). The patients/participants provided their written informed consent to participate in this study. Use of animal and the experimental procedures were in accordance with the Institutional Animal Care and Use Committee guidelines and approved by the Animal Care Committee of Jiangsu Vocational College of Medicine (project number: XMLL-2022-811).

## Data Availability

All data generated or analyzed in this study were included in the main document and Supplementary Materials of this article. Source data are provided with this paper. Any additional information is available upon request to the corresponding author (W.C., wangsencao@nju.edu.cn).
