# Supplementary material for: Epigenetic Suppression of RASAL1 by HDAC3 and Cofactor YY1 Promotes Fibroblast–Myofibroblast Transition and Renal Fibrosis
Source: Research (Wash D C). 2026 Jan 29;9:1073. doi: 10.34133/research.1073 (PMC12852569; doi:10.34133/research.1073)
Supplement: Supplementary 1 — Fig. S1 Table S1 [file research.1073.f1.zip › 25-12-3 Supplementary Figure .docx]

Supplementary Materials for

**Epigenetic Suppression of RASAL1 by HDAC3 and Cofactor YY1 Promotes Fibroblast-Myofibroblast Transition and Renal Fibrosis**

**Supplementary Figure S1**


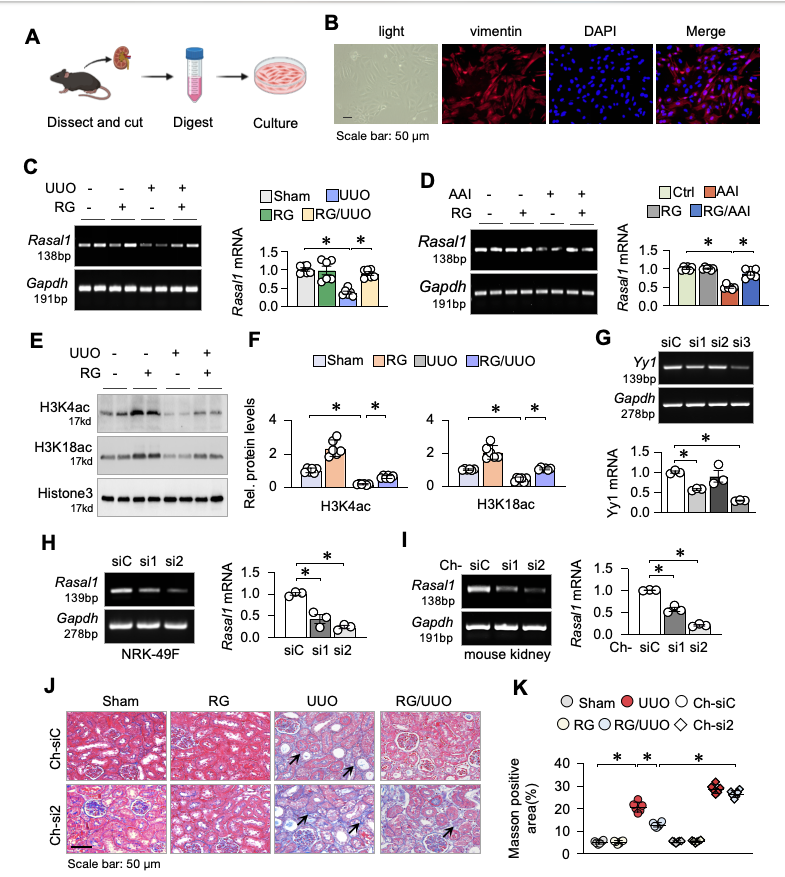


1. Schematic diagram of isolation of mouse primary renal fibroblasts (PRF) cells. **(B)** Representative immunofluorescence staining of vimentin in PRF isolated from mice. C57BL/6 mice were subjected to Sham/UUO for 7 days, or Control/AAI for 14 days treated with or without RGFP966 (RG, 10 mg/kg, i.p. daily, n=6). RT-PCR of *Rasal1* mRNA in renal tissues from Sham, RG, UUO and RG/UUO group mice **(C)** or from Control (Ctrl), RG, AAI and RG/AAI group mice **(D)**. The right panels were the quantifications. **(E)** Western blots of Sham, RG, UUO and RG/UUO renal tissues using site-specific antibody to acetylated histone 3 lysine 4 (H3K4ac) and H3K18ac. **(F)** The quantifications of protein expression in E. Data were presented as means ± SEM, **P <* 0.05, n = 6, two-way ANOVA. **(G)** NRK-49F cells were transfected with control lentivirus (siC) and three YY1 knockdown lentiviruses (si1, si2 and si3) for 48 h. RT-PCR was performed on cell lysates to measure Yy1 mRNA levels. The lower panel was the quantification. **(H)** NRK-49F cells were treated with small interfering RNA control (siC), siR-Rasal1-1 (si1) or siR-Rasal1-2 (si2) for 48h. RT-PCR of the cell lysates were assayed for *Rasal1* mRNAs. The right panel was the quantification. Data were presented as means ± SD of three repeated experiments. **P* < 0.05, one-way ANOVA. **(I)** C57BL/6 mice were treated with cholesterol-siRNA-Control (Ch-siC), Ch-siR-Rasal1-1 (Ch-si1) or Ch-siR-Rasal1-2 (Ch-si2) once by intravenous injection, three mice in each group. Seven days later, RT-PCR was performed on the mouse renal tissues for *Rasal1* mRNAs. The right panel was the quantification. Data were presented as means ± SEM. **P* < 0.05, one-way ANOVA. **(J)** Mice receiving Ch-siC or Ch-si2 were subgrouped into Sham, RG, UUO, and RG-treated UUO (n=6). Representative photomicrographs of kidney sections stained by Masson’s trichrome. The dark arrows indicate fibrotic areas. **(K)** Quantification of J. Data were presented as Scatter plot. **P* < 0.05, three-way ANOVA followed by Tukey’s post-hoc test.
