# Supplementary material for: Epigenetic Suppression of RASAL1 by HDAC3 and Cofactor YY1 Promotes Fibroblast–Myofibroblast Transition and Renal Fibrosis
Source: Research (Wash D C). 2026 Jan 29;9:1073. doi: 10.34133/research.1073 (PMC12852569; doi:10.34133/research.1073)
Supplement: Supplementary 1 — Fig. S1 Table S1 [file research.1073.f1.zip › Table 1 Patient information.docx]

**Table 1: Patient Information**

| Group | No. | Sex | Age (y) | Primary Disease Diagnosis | CKD  Stage | Fibrosis  (% area) | SCR (μmol/L) | BUN (mmol/L) |
| --- | --- | --- | --- | --- | --- | --- | --- | --- |
| Mild  Kidney Injury | 1 | F | 34 | IgA Nephropathy | 2 | ~5% | 89.6 | 7.17 |
|  | 2 | M | 14 | IgA Nephropathy | 1 | ~10% | 60.5 | 4.31 |
|  | 3 | F | 34 | Chronic nephritis Syndrome | 1 | ~10% | 59 | 4.09 |
|  | 4 | M | 21 | Nephritis Syndrome | 1 | <5% | 63.7 | 6.9 |
|  | 5 | M | 61 | Nephritis Syndrome | 2 | ~5% | 91.7 | 7.27 |
|  | 6 | M | 47 | HBV-Associated Glomerulonephritis | 1 | <5% | 80 | 11.09 |
| CKD/Severe Kidney Injury | 1 | M | 53 | Renal Insufficiency | 3b | 30-35% | 159.7 | 7.89 |
|  | 2 | M | 37 | IgA Nephropathy | 3a | ~20% | 132.8 | 6.08 |
|  | 3 | M | 74 | Nephrotic Syndrome | 4 | ~30% | 230.7 | 15.81 |
|  | 4 | M | 50 | Hypertensive Nephropathy | 3a | 20-25% | 129.8 | 7.24 |
|  | 5 | M | 52 | Nephrotic Syndrome | 3a | 45-50% | 137.9 | 4.86 |
|  | 6 | M | 31 | Diabetic Nephropathy | 3a | 45-50% | 168.8 | 6.21 |
|  | 7 | F | 37 | IgA Nephropathy | 3a | 40-45% | 105.7 | 7.22 |
|  | 8 | M | 28 | Chronic Kidney Disease Stage 3 | 3b | 40-50% | 182.6 | 8.76 |
|  | 9 | F | 65 | Acute Renal Failure | 5 | ~35% | 373.5 | 17.95 |
|  | 10 | M | 67 | ANCA-Associated Vasculitis | 4 | 25-30% | 202.2 | 7.62 |
