# Supplementary figures and images for: Epigenetic Suppression of RASAL1 by HDAC3 and Cofactor YY1 Promotes Fibroblast–Myofibroblast Transition and Renal Fibrosis
Source: Research (Wash D C). 2026 Jan 29;9:1073. doi: 10.34133/research.1073 (PMC12852569; doi:10.34133/research.1073)

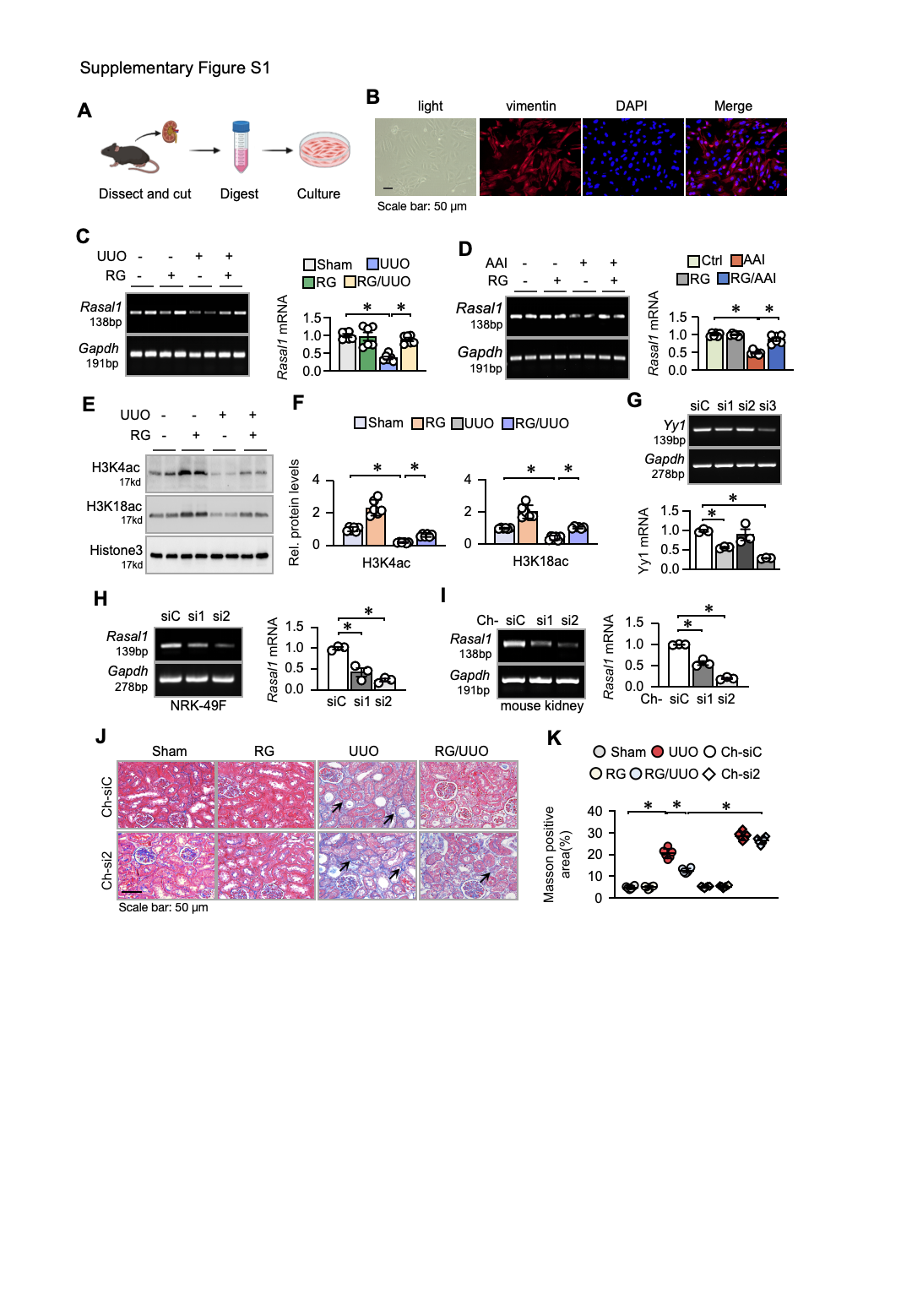

Supplement: Supplementary 1 — Fig. S1 Table S1 [file research.1073.f1.zip › 25-12-1 supplementary figure.tif]
